# Variants near *CHRNA3/5* and *APOE* have age- and sex-related effects on human lifespan

**DOI:** 10.1038/ncomms11174

**Published:** 2016-03-31

**Authors:** Peter K. Joshi, Krista Fischer, Katharina E. Schraut, Harry Campbell, Tõnu Esko, James F. Wilson

**Affiliations:** 1 Centre for Global Health Research, Usher Institute for Population Health Sciences and Informatics, University of Edinburgh, Teviot Place, Edinburgh EH8 9AG, Scotland; 2 Estonian Genome Center, University of Tartu, Riia 23b, 51010 Tartu, Estonia; 3 Centre for Cardiovascular Sciences, Queen's Medical Research Institute, University of Edinburgh, Royal Infirmary of Edinburgh, Little France Crescent, Edinburgh EH16 4TJ, Scotland; 4 Division of Endocrinology and Center for Basic and Translational Obesity Research, Boston Children's Hospital, Cambridge, Massachusetts 02141, USA; 5 Program in Medical and Population Genetics, Broad Institute, Cambridge Center 7, Cambridge, Massachusetts 02242, USA; 6 Department of Genetics, Harvard Medical School, 25 Shattuck St, Boston, Massachusetts 02115, USA; 7 MRC Human Genetics Unit, Institute of Genetics and Molecular Medicine, University of Edinburgh, Western General Hospital, Crewe Road, Edinburgh EH4 2XU, Scotland

## Abstract

Lifespan is a trait of enormous personal interest. Research into the biological basis of human lifespan, however, is hampered by the long time to death. Using a novel approach of regressing (272,081) parental lifespans beyond age 40 years on participant genotype in a new large data set (UK Biobank), we here show that common variants near the apolipoprotein E and nicotinic acetylcholine receptor subunit alpha 5 genes are associated with lifespan. The effects are strongly sex and age dependent, with *APOE* ɛ4 differentially influencing maternal lifespan (*P*=4.2 × 10^−15^, effect −1.24 years of maternal life per imputed risk allele in parent; sex difference, *P*=0.011), and a locus near *CHRNA3/5* differentially affecting paternal lifespan (*P*=4.8 × 10^−11^, effect −0.86 years per allele; sex difference *P*=0.075). Rare homozygous carriers of the risk alleles at both loci are predicted to have 3.3–3.7 years shorter lives.

## Abstract (web summary)

Understanding the genetic influences on human aging requires a large number of subjects for a study of sufficient power. Here, Jim Wilson and colleagues use information on parental ages at death to show that common variants near the genes for apolipoprotein E and nicotinic acetylcholine receptor subunit alpha 5 are associated with longer lifespan.

## Graphical abstract

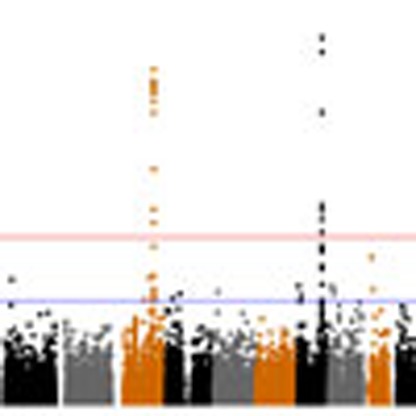

Being alive is the simplest, summary measure of overall health. Thus the genomics of longevity offer the prospect of illuminating system-wide components of health that are not related to disease and biological systems involved in both frailty and healthy ageing. Genetic factors are suggested to account for up to 16–25% of the variation in long-livedness^1^,^2^. However, whereas studies in model organisms have revealed signalling pathways with profound influences on lifespan^3^, only one locus has consistently been associated with human survival: *APOE*^4^,^5^,^6^. Efforts to discover additional age-related genetic loci are impeded by difficulties in recruiting very large prospective cohorts with lifespan and genomic data that are required to give sufficient power to detect the expected small effects associated with this complex trait. Although many population cohorts can capture family history, subjects recruited in middle age will have aged or deceased parents. UK Biobank^7^ (UKB) is a particularly rich resource in this context: half a million people were recruited aged 40–69 years and genome-wide genotypes are presently available for 152,732 subjects. The first genetic association analysis in UKB for lung function has recently been published^8^. Hundreds of thousands of parents of UKB participants were deceased at the time of baseline measurements. The use of phenotyped (but not genotyped) relatives of subjects who have been genotyped to conduct association analyses between phenotype and probabilistically inferred genotype, generalizes the approach of Wacholder *et al.*^9^ from Mendelian traits to quantitative traits and provides a step change in power, applicable to all late onset conditions, where direct genotyping may be problematic. Here we use this approach to show that common variants near the apolipoprotein E and nicotinic acetylcholine receptor subunit alpha 5 genes are associated with lifespan in a sex- and age-dependent manner. The *APOE* ɛ4 allele differentially influences maternal lifespan (*P*=4.2 × 10^−15^, effect −1.24 years of maternal life per imputed risk allele in parent), and a locus near *CHRNA3/5* differentially affects paternal lifespan (*P*=4.8 × 10^−11^, effect −0.86 years per allele). The ∼0.3% of Europeans who are homozygous carriers of the risk alleles at both loci are predicted to have 3.3–3.7 years shorter lives. Thus the major genetic determinants of lifespan are both very pleiotropic disease susceptibility genes.

## Results

### Discovery cohort

**Figure 1:**
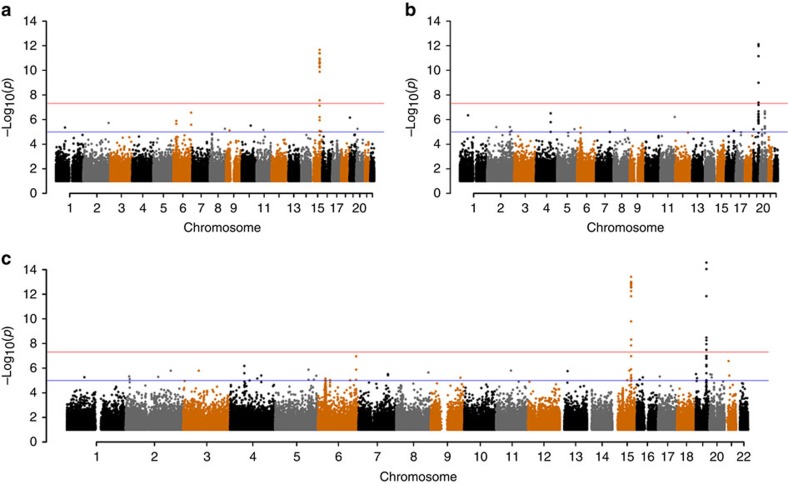
Genome-wide association with parental lifespan. Manhattan plots are presented for the discovery analysis in genetically British individuals (**a**) for fathers, (**b**) for mothers, (**c**) for meta-analysis of parents. In each case the trait is the Martingale residuals of the Cox proportional hazards model of parental lifespan. rs429358 and rs10519203 explain, respectively, 0.026/0.068% and 0.042/0.012% of the variance of the Martingale residuals in fathers/mothers.

**Figure 2:**
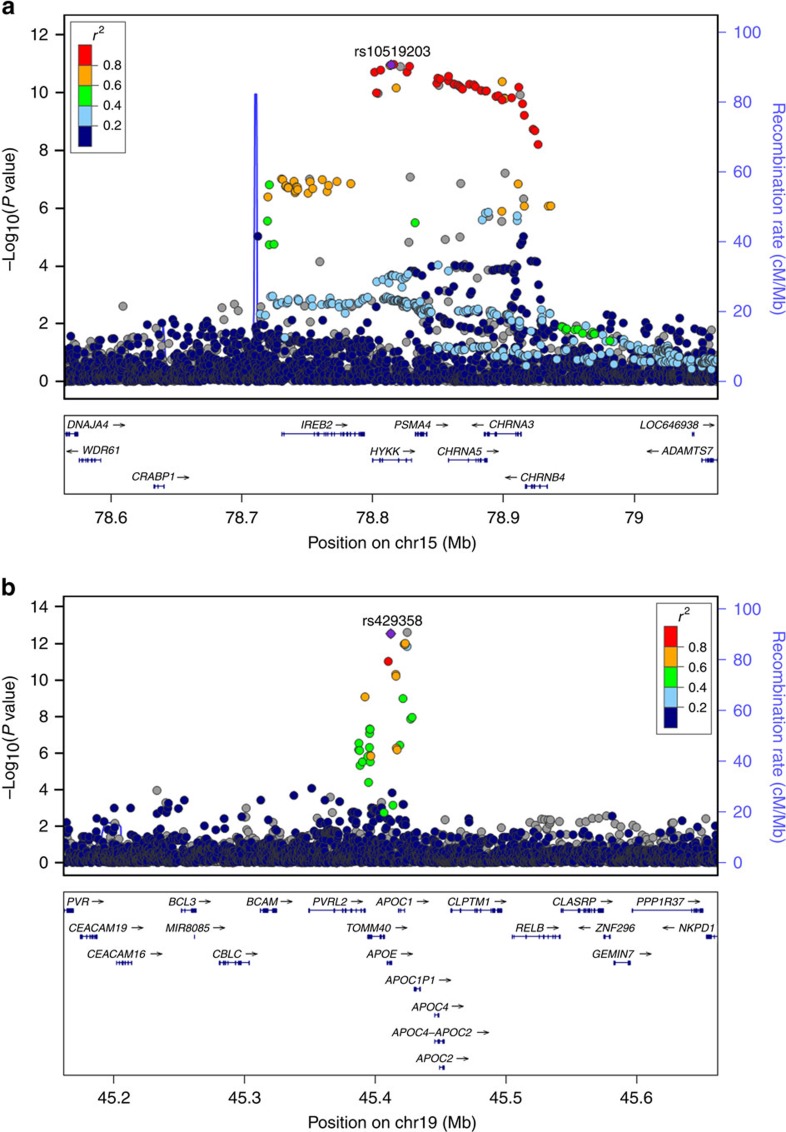
**Locus zoom** **plots for the two robust associations with parental lifespan.** (**a**) *CHRNA3/5* region and paternal lifespan, (**b**) *APOE* region and maternal lifespan.

In the discovery phase genome-wide association (GWAS), using rank-normalized Martingale residuals of survivorship for 116,425 UKB subjects of unambiguous British genetic ancestry, we discovered 35 single nucleotide polymorphisms (SNPs) (Supplementary Table 1) with a *P* value <10^−6^ for association in meta-analysis of paternal and maternal lifespans. However, only two loci reached genome-wide significance (*P*<5 × 10^−8^), on chromosome 15 and 19 (Fig. 1). Several SNPs are associated with lifespan in each region, the most significant being rs429358 in *APOE* at chromosome 19q13 and rs10519203 in a locus including *HYKK*, *PSMA4*, *CHRNA5*, *CHRNA3* and *CHRNB4* at chromosome 15q24 (Fig. 2). However, the chromosome 15 association, which we refer to hereafter as *CHRNA3/5*, appears much stronger in men than in women and conversely the *APOE* association is much stronger in women than men (Fig. 1). Q–Q plots (Supplementary Fig. 1a–c) show no inflation, suggesting that there are no issues with relatedness, population structure or other confounding factors. Conditioning on both sentinel SNPs removed all other signals in these regions and the distribution of *P* values appeared consistent with chance (Supplementary Fig. 1d); there was also no evidence of associations at *FOXO3* and 5q33, recently associated with nonagenarian subjects^6^,^10^.

Under the full Cox model in the discovery cohort (that is, genotype effect fitted simultaneously with other covariates), the per allele hazard ratios across both parental sexes were 1.115 (*P*=6.29 × 10^−19^) for the C allele at rs429358 and 1.057 (*P*=1.95 × 10^−13^) for the G allele at rs10519203 (Table 1). However, the effect sizes in each parental sex differed strongly. The survival reduction due to rs429358 at *APOE* was 0.79 and 1.24 years per risk allele and due to rs10519203 near *CHRNA3/5* was 0.86 and 0.60 years in fathers and mothers, respectively (Table 1; the *P* value for a difference in hazard ratio between the sexes was 0.011 for rs429358 and 0.073 for rs10519203; two-sided *t*-test of contrast). Bivariate analysis also showed that association is mediated through rs429358, not rs4420638 near *TOMM40* (in high linkage disequilibrium, *r*^2^=0.71): bivariate log_e_ hazard ratio (±1 s.e.) for mothers in the discovery cohort were 0.067 (±0.017) and 0.005 (±0.018), respectively.

### Replication

We sought replication in three data sets, two from non-genetically British sub-cohorts of UKB using parental lifespans, and one from the Estonian Biobank using participant survival. Hazard ratios in the three replication cohorts were all directionally consistent, and fell within two s.e'.s of the estimate in the discovery cohort. After meta-analysis of the three replication studies (not including the discovery cohort), the *P* values for association were 0.0020 for rs429358 in *APOE* and 6.8 × 10^−6^ for rs10519203 near *CHRNA3/5*, and estimated effect sizes were again consistent with the discovery cohort (Table 1). In an overall meta-analysis of the discovery and replication sets, *P* values were 1.15 × 10^−20^ for rs429358 and 1.8 × 10^−17^ for rs10519203.

### Age-dependent effects

To investigate whether effects varied by age at death, we compared allele hazard ratios at ages-of-death of 40–75 and over 75, very broadly the mean age across men and women, alive and dead. The estimated hazard ratio for rs429358 in *APOE* was larger at older ages: 1.09 in younger mothers and 1.20 in older mothers (the more affected sex), with 0.2 life years lost before age 75 years, but 1.1 years beyond it (Table 2). The estimated hazard ratio for rs10519203 near *CHRNA3/5* was smaller at older ages: 1.10 in younger fathers and 1.03 in older fathers (the more affected sex), with 0.5 life years lost before age 75 years, but only 0.1 years beyond it. The *P* value for the difference in log hazard ratios at younger and older ages, is <0.006 in the more affected sex for both variants, and although directionally suggestive, is not significant (*P*>0.05) for the less affected sex for both alleles, (two sided t-test of contrast in effect size, in both cases).

The effect sizes are relatively large, conferring between 0.6–1.2 years less life per allele (by sex) for both loci. Homozygous carriers of the risk alleles at both loci (around 0.27% of the population) are predicted to have 3.3–3.7 years shorter lives compared to homozygotes carrying the protective variants.

## Discussion

We have shown that common variants at two loci are associated with lifespan, and these effects are strongly age- and sex-related. The APOE* ɛ4 allele increases in particular the risk of mortality for older females, whereas a variant near *CHRNA3/5* has stronger effects on young to middle-aged men.

Apolipoprotein E is a receptor-binding ligand on the surface of chylomicrons, intermediate and low-density lipoprotein particles, and is involved in uptake and redistribution of lipids. It is widely expressed, most strongly in the liver, brain and retina. The signal we observe is driven by rs429358, a non-synonymous Cys112Arg variant, which defines the ɛ4 allele and which has not previously been shown to be the causal variant influencing lifespan. Previous findings of association of longevity with rs4420638 near *TOMM40* (refs 5, 6), appear to arise from linkage disequilibrium with rs429358 (not genotyped in the prior work). The *APOE* locus and in some cases specifically the ɛ4 allele have been associated with an array of age-related conditions and traits, including Alzheimer's dementia^11^, age-related cognitive decline^12^, total and low-density lipoprotein cholesterol and triglycerides^13^, C-reactive protein^14^, coronary disease risk^15^ as well as centenarianship^16^ and survival to 90 years^5^. The association we observe with lifespan is potentially due to the combined impact of the cognitive and cardiometabolic effects on mortality. There has been debate as to whether *APOE* primarily influences mortality across the spectrum of ages at death (frailty) or survival to old age (longevity). In stratified analyses for parental deaths before and after 75 years, we see a much stronger effect of ɛ4 on dying after 75 years, for both mothers and fathers, providing strong additional evidence for an effect on survival to >90 years^4^,^5^,^6^.

Whereas *APOE* influences the lifespan of fathers and mothers, we observe robust evidence for a stronger effect in mothers. No sex-specificity has been reported for the effects on lipids, but there is evidence for a stronger effect of *APOE* on Alzheimer's risk in females^17^. The effect on cognitive function is also much stronger in females^18^ and longitudinal analyses confirm the mortality effect is strongest for female lifespans of about 70–95 years^19^.

Smoking is the single-most important cause of premature death in the developed world, increasing the risk of many diseases including lung cancer, coronary heart disease, chronic obstructive pulmonary disease and emphysema. The *CHRNA5-CHRNA3-CHRNB4* locus encodes various nicotinic acetyl choline receptor subunits, ion channels that mediate fast signal transduction at synapses. Various SNPs in our associated interval have been associated with smoking quantity^20^, nicotine dependence^20^, lung cancer^21^, chronic obstructive pulmonary disease^22^, peripheral arterial disease^20^, alcohol dependence (independent of smoking status^23^) and schizophrenia^24^. The region is modestly associated (*P*=1 × 10^−4^) with airway obstruction in never smokers^25^, and the effects on lung cancer and chronic obstructive pulmonary disease remain after adjusting for self-reported smoking intensity. Thus it remains unclear if these effects are mediated entirely by smoking behaviour, if they also involve an increased vulnerability to the harmful effects of smoking or if there is a smoking-independent effect. The recently discovered association with exhaled carbon monoxide levels^26^ suggests aspects of cigarette smoke exposure not captured by common measures of smoking quantity and thus the residual associations may still be due to genetic effects on smoking exposure. Interestingly a potential association (*P*=6 × 10^−7^) has been reported for serum albumin levels in a large meta-analysis^27^, a known biomarker of all-cause mortality after 5-year follow-up^28^. Variants in linkage disequilibrium (LD) with the sentinel SNP are also associated with cotinine^29^ (the principal metabolite of nicotine), 1-methylurate^29^ (a major metabolite of theophylline, used to treat chronic obstructive pulmonary disease), and lathosterol^29^ (an indicator of whole body cholesterol synthesis). There is no reported evidence of sex differentiation in the known associations but we observe a much stronger effect of rs10519203 for younger paternal deaths, consistent with the higher prevalence of smoking among men of that generation^30^.

The relative importance of frailty genes, affecting the age-specific susceptibility to death, versus longevity genes, which promote long life, has been much debated^31^. The *CHRNA3/5* locus is the first true frailty gene to be identified, as the mortality of carriers of the risk variant decreases to nearly that of non-carriers with advancing age. This can be explained by the longer-lived carriers of the G allele at rs10519203 possessing other genetic or environmental factors that compensate for the risk (for example, never smoked). In contrast *APOE* has a stronger effect on mortality for the older deaths.

We did not replicate the recently published chromosome 5q33 or *FOXO3* associations with survival to 90 years of age^6^,^10^ with *P* values over 0.02 for 87 SNPs across 400 kb, despite considerable power to do so. Possible explanations include that these loci influence longevity in a way that is diluted when considering deaths from 40 onwards, or that they are false positive associations.

In spite of the similar magnitude of effects for *CHRNA3/5* and *APOE* in our analysis of >190,000 parental deaths, the *CHRNA3/5* association was not seen in the largest published nonagenarian analyses^6^,^10^, probably because the association with death is very weak after 75 years, and is driven mostly by younger male deaths, many of which might have already occurred before participation in the population-based studies analysed.

The adverse effects on lifespan of the two associated loci have both likely been hidden from natural selection, *CHRNA3/5* because its effect is mediated through the modern risk of nicotine dependence and *APOE*, because its effect occurs long after child-bearing age. The absence of evidence for other loci affecting lifespan (either in terms of more genome-wide significant associations—Fig. 1 or an enrichment of suggestive associations beyond that expected by chance—Supplementary Fig. 1d), is difficult to interpret as rarer variants or those with weaker effects might be discovered in even larger analyses, such as with the entire UKB sample.

Nonetheless, we have shown that even a trait as complex as parental lifespan is amenable to GWAS using the power provided by hundreds of thousands of data points. The two lifespan loci discovered here both contain very pleiotropic disease susceptibility genes, influencing risk for multiple disorders that cause most deaths in the developed world. The major genetic risk factors for dying appear to represent the sum total of genetic effects on mortality mediated by the different diseases they influence. The large number of parental lifespans analysed provide robust evidence for large sex- and age-differentiated effects of both *APOE* and *CHRNA3/5* variants, which merit further investigation. Thus, whereas parental traits may be of interest in diverse spheres of biology, lifespan, as the ultimate trait, benefits more than any other from using the kinship between offspring and parent to increase power for association.

## Methods

### Outline

Our study closely followed standard methods for GWAS meta-analysis studies of a quantitative trait (GWAMA)^32^, with human lifespan as the phenotype, with the following exceptions. First, the phenotyped subjects were parents of participants in UKB. Parental phenotypes were regressed on offspring genotypes, in effect imputing parent genotype from offspring. The effects measured are thus of offspring genotype on parental phenotype and the expected allelic doses in the parental generation are thus half the measured doses (in offspring). Effect estimates per parental allele are thus twice that of offspring allele, which is what we report unless otherwise stated. Second, as all subjects were genotyped on only two highly overlapping arrays, focus was on array, rather than imputed genotypes.

### UK Biobank

UKB participants were recruited from the general UK population across 22 centres between 2006 and 2010 (ref. 7). Subjects were aged 40–69 at baseline, underwent extensive phenotyping by questionnaire and clinic measurements, and provided a blood sample. All participants gave written informed consent and the study was approved by the North West Multicentre Research Ethics Committee. UKB has Human Tissue Authority research tissue bank approval, meaning separate ethical approvals are not required to use the existing data. Genotyping is in progress, with a wave 1 public release in June/July 2015. Data access to UKB was granted under MAF 8304. Phenotypes and genotypes were downloaded direct from UKB. In total, 502,664 subjects had phenotypic information available, of whom 152,732 had been genotyped. Parental ages and whether they were alive at time of interview were reported in the UKB questionnaire.

### Estonian biobank

The Estonian Biobank^33^ is the population-based biobank of the Estonian Genome Center at the University of Tartu (EGCUT). A total of 51,380 volunteer participants (aged 18–103) were recruited between 2002 and 2011. The cohort included ∼5% of the population from all counties of Estonia. At recruitment, the participants completed an extensive questionnaire on health, lifestyle and genealogy and provided a blood sample. A broad informed consent enables regular linkage of the data with the Estonian mortality registry providing accurate data on participants' mortality and causes of death. As of April 2014, there were 2,663 mortalities registered in the cohort, with mean follow-up time for the surviving being 6 years. In total, DNA samples from >16,000 participants have been genotyped with various genome-wide arrays. In 2011, the subset of individuals selected to be genotyped with the Illumina OmniExpress chip, intentionally included 1,200 individuals who had died by that time, as well as 500 women and 250 men who were 80 years old or older at that time. The rest of this genotyped sample (in total 7,950 subjects after removing close relatives) consists of random population controls.

### Genotyping

UKB participants were genotyped on two slightly different arrays and quality control was performed by UKBiobank. In brief, ∼50,000 were genotyped as part of the UK BiLEVE study using a newly designed array, with the remaining samples (∼100,000) genotyped on an updated version (UK Biobank Axiom array), both manufactured by Affymetrix (96% of SNPs overlap between the arrays). Samples were processed and genotyped in batches (used as covariates to control for confounding due to batch effects). In brief, SNPs or samples with high missingness, multi-allelic SNPs and SNPs with batchwise departures from Hardy–Weinberg equilibrium were removed from the data set. Analysis was restricted to the 753,488 autosomal SNPs passing these filters^34^. After quality control, genotypes were available for 152,732 subjects. UKB provided 15 principal components of genetic relatedness (Biobank field id 22,009) and a binary assessment of whether subjects were genomically British (biobank field id 22,006), based on principal components analysis of their genetic data.

Imputed data were prepared by UKB and used for fine-mapping signals in two genomic regions of interest. In summary, autosomal phasing was carried out using a version of SHAPEIT2 (ref. 35) modified to allow for very large sample sizes. Imputation was carried out using IMPUTE2 (ref. 36) using the merged UK10K and 1,000 Genomes Phase 3 reference panels to yield higher imputation accuracy of British haplotypes. The result of the imputation process is a data set with 73,355,667 SNPs, short indels and large structural variants^37^ . Our analysis for the Chr15 region (77,826,182–79,826,157 bp) included 54,767 variants and 54,474, respectively, for the Chr19 region (44,422,982–46,422,870 bp).

The Estonian Biobank genotypes were from the Illumina OmniExpress array. A total of, 647,357 autosomal SNPs were available for 7,950 subjects. Standard quality control practices were followed, including removing SNPs and individuals with high missingness, samples with mismatching genomic and self-declared gender, ethnic outliers, duplicates, cryptic relatives, SNPs out of Hardy–Weinberg equilibrium. Imputation followed similar workflow to UKB, using SHAPEIT2, IMPUTE2 and the 1,000 Genomes Phase 3 reference panel.

### UKB quality control

For the genotyped subjects, phenotypic quality control proceeded as follows. Subjects had completed a questionnaire which asked, *inter alia,* whether parents were alive or dead, the parents' ages and whether the subject was adopted. Parent deaths prior to the age of 40 were excluded to reduce the effect of accidents and war. Our phenotype was thus survival from the age of 40. Subjects who were adopted (2,296), whose parents both died before age 40 or for whom no parental ages/alive status were recorded (3,278), whose parent ages were clearly invalid (>115; *n*=2), or had non-useful (small or vague) ethnicity recorded (7,065) or had a missing covariate (181) were excluded, leaving 139,910 participants with complete father or mother data for analysis.

Of these, 116,279 were self-identifying British and were assessed as genetically British by UKB; these were the subjects analysed in the discovery phase. Supplementary Table 2 presents the mean and maximum parental ages by status for these subjects.

In the UKB part of the replication phase, we analysed subjects not included in the discovery phase, that is, subjects who had not self-identified as ‘British' or UKB had determined as not unambiguously genetically British. Self-declared ethnicities that had fewer than 300 subjects (‘Asian', ‘Bangladeshi', ‘black', ‘mixed') or were ambiguous (‘any other', ‘prefer not to say', ‘white'), were not analysed. This resulted in 23,631 subjects across seven sub-populations: self-declared British—but not unambiguously genomically British 16,218; African 681; Caribbean 962; Chinese 338; Indian 1,245; Irish 3,819; Pakistani 368. More details on the counts of subjects flowing through quality control are available in Supplementary Table 3.

### Association testing

Although our analysis followed the broad strategy of best practice GWAMA, the unique characteristics of our data set and, to a lesser extent, trait meant we modified the design slightly. All subjects were genotyped on only two, very similar, arrays. This meant we could perform GWAS directly on array SNPs, without losing the majority of SNPs due to missingness, an issue usually addressed through imputation. The computational burden of fitting a full Cox model and GWAS one SNP at a time across all array SNPs was infeasible for 116,279 subjects. So, we first calculated Martingale residuals^38^ for survivorship in the Cox proportional hazards model (CPHM)^39^ excluding genotype using the R package survival and then used PLINK 1.9 (ref. 40) to perform a GWAS scan at array SNPs for these residuals. Manhattan plots were created in R using the package qqman^41^. For the two clearly associated regions, we then created locus zoom plots using imputed genotypes and the Martingale residuals (the discovery phase). The top array SNP for mothers and fathers combined in each unambiguously associated region was then analysed using a full CPHM simultaneously modelling array genotype and other covariates, allowing us to determine *P* values directly and calculate allelic effects on the more natural scales of hazard ratio and life years (verification phase). Finally, the association between the top array SNPs and lifespan was tested in three other populations (replication phase).

Association testing was conducted under the following CPHM,


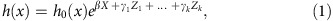


with *h*_0_(*x*) being the baseline hazard given the parental age and dead/alive status, *X* the non-reference allele count of the marker and *Z*_1_, …, *Z*_*k*_ the other covariates in the model: subjects' (not parental) sex, indicators of assessment centre and genotyping batch, Townsend deprivation index (a measure of socio-economic status), and 15 principal components of genetic relatedness for all 152,732 considered together.

Where shown, results for fathers and mothers combined were calculated using inverse-variance meta-analysis on the log hazard ratios.

In the discovery phase (using unambiguously genetically British), Martingale residuals of the Cox model (without genotype) were calculated using the R package ‘Survival'. The Martingale residuals^38^ are defined as,


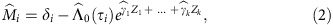


where *δ*_*i*_ and *τ*_*i*_ are the event indicator (1—died, 0—survived at the end of follow-up) and follow-up time of the *i*th individual, 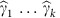 are the estimates of the coefficients of the covariates *Z*_1_ … *Z*_*k*_ in the model from equation (1), omitting the genetic markers. In case of non-zero *β* that model 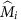 should have a linear association with the marker score *X*_*i*_. These residuals were subsequently rank-normal transformed to give a normally distributed survival score for each subject. These scores were then tested for association with genotype using PLINK. This process was then repeated but conditioned on the two top SNPs (rs429358 and rs10519203), to determine if there was evidence of additional allelic effects local to their regions.

In the verification phase the two top SNPs were analysed singly using the full Cox model (including genotype), in R, to give hazard ratios for additive allelic effect and with Kaplan–Meier survival curve^42^ being used to calculate expected lifetime for each genotype. The bivariate analysis of rs4420638, simultaneously fitted the three SNPs.

In the replication phase, rs429358 and rs10519203 were analysed under the full Cox model. In UKB each sub-population was analysed separately (self-declared British—but not unambiguously genomically British; African, Caribbean, Chinese, Indian, Irish, Pakistani) for the effect of allele count on log hazard ratio. Results were combined for UKB non-self-declared British using a two-sided test and inverse variance meta-analysis.

We performed an enhanced replication in the Estonian Biobank using participant lifespans that simultaneously tested whether the effects on parental lifespan translated into an influence on survival in a prospective cohort. All-cause mortality was analysed using Cox modelling in 5,196 individuals including 1,499 deaths from ages 40 to 103, over a mean follow-up time of 6 years, using the first three principal components of genetic relatedness as covariates, in addition to the effect allele count of the sentinel SNPs. Three models were fitted, a sex-stratified model and sex-specific models for males and females. Given the data structure, where an excess number of mortalities were included in the genotyped set compared with the full cohort, baseline hazard and survival estimates are not valid, but hazard ratios are. As a sensitivity analysis, a case–cohort analysis was performed^43^, resulting in identical effect estimates and *P* values.

The replication cohorts' results (Estonian Biobank, self-declared British and self-declared non-British) were then combined using inverse variance meta-analysis.

Finally, for the unambiguously genomically British samples only (the discovery phase), we looked at the effect of the risk SNPs over two separate age ranges 40–75 and >75 (approximately the mean age at death). To do this, the same Cox models were rerun, but for the first age range, upper age was truncated at 75, and subjects who lived past aged 75 were recorded as survivors. For the second age range, only subjects who had survived to age 75 were entered into the analysis, survival to age 75 was thus complete, and the analysis only examined survival beyond age 75.

The expected allelic effect of one allele in offspring is 0.5 alleles in parents. This can be seen, for an allele (A1) with frequency *p*, because

- if the offspring is homozygous for the other allele (A0), the expected dosage of A1 in a parent is *p* (that is, the probability that the non-inherited allele is A1).
- if the offspring is homozygous A1 the expected dosage in the parent is 2−*q*=1+*p*.
- if the offspring is heterozygous, the expected dosage in a parent is the average of the previous two values.

Measured effects of offspring genotypes, other than the variances explained in Fig. 1 have therefore all been doubled, to give measurement on the natural scale—the effect on parents' lifespan of one allele in parents.

## Additional information

**How to cite this article:** Joshi, P. K. *et al.* Variants near *CHRNA3/5* and *APOE* have age- and sex-related effects on human lifespan. *Nat. Commun.* 7:11174 doi: 10.1038/ncomms11174 (2016).

## Supplementary Material

Supplementary Information Supplementary Figure 1, Supplementary Tables 1-3

## Figures and Tables

**Table 1:** Association of paternal and maternal lifespan in the UK Biobank discovery analysis.

**Population**	**Parent**	**Lives**	**Deaths**	**Beta**	**HR**	**s.e.**	***P*** **value**	**Years**
								
*rs429358, chr19:45411941 C allele APOE*								
UKB: Genet. British	Father	73,100	55,568	0.0805	1.084	0.0165	1.08 × 10^−6^	−0.79
UKB: Genet. British	Mother	75,576	45,254	0.1426	1.153	0.0182	4.22 × 10^−15^	−1.24
Discovery	Both	148,676	100,822	0.1086	1.115	0.0122	6.29 × 10^−19^	
*P* sex effect							0.011	
UKB: declared British	Both	20,874	13,979	0.0341	1.035	0.0337	0.31	
UKB: other origins	Both	11,771	7,483	0.1146	1.121	0.0488	0.0189	
Estonian Biobank	Offspring	5,196	1,499	0.1190	1.120	0.0560	0.034	
Replication		37,841	22,961	0.0716	1.074	0.0248	0.0020	
Overall		186,517	123,783	0.1014	1.107	0.0110	1.15 × 10^−20^	
								
*rs10519203, chr15:78814046 G allele CHRNA3/5*								
UKB: Genet. British	Father	111,025	85,182	0.0675	1.070	0.0103	4.83** ×** 10^−11^	−0.86
UKB: Genet. British	Mother	115,003	69,796	0.0403	1.041	0.0113	3.84** ×** 10^−4^	−0.60
Discovery	Both	226,028	154,978	0.0552	1.057	0.0076	1.95 × 10^−13^	
*P* sex effect							0.075	
UKB: declared British	Both	31,349	21,309	0.0675	1.070	0.0206	0.0010	
UKB: other origins	Both	14,223	9,308	0.0516	1.053	0.0335	0.124	
Estonian Biobank	Offspring	5,196	1,499	0.1010	1.130	0.0390	0.0100	
Replication		50,768	32,116	0.0695	1.072	0.0160	6.82 × 10^−6^	
Overall		276,796	187,094	0.0579	1.060	0.0069	1.82 × 10^−17^	

*P* values are given for the discovery analysis allele dose effect for the sentinel SNP at each locus, separately for maternal and paternal lifespans, and the meta-analysis of both. Discovery was carried out in the genetically British subset of UKB, with replication for parental lifespans in (a) the self-declared British who did not meet the strict UKB definition of genetically British (declared British), (b) UKB participants of other origins (declared and genetic) who were analysed as separate ethnic groups and then meta-analysed and (c) with participant survival in participants in the Estonian Biobank. The effects in UKB reflect imputation of 0.5 parental allelic doses from 1 offspring allelic dose. Beta is the effect size from the full Cox model, HR is hazard ratio, years gives the reduction in lifespan in years for a (parental generation) individual carrying one copy of the variant. The number of observations for rs429358 is much lower than for rs10519203, because this locus was not included in the UK BiLEVE array used to genotype 50,000 subjects, nevertheless it was the most significant variant in the region. The chromosome co-ordinates use GRCh37. Genet. British are genetically British.

**Table 2:** Associations with lifespan are age-dependent.

**Parent**	**Age range**	**Lives**	**Deaths**	**Beta**	**HR**	**s.e.**	***P*** **value**	**Years**
								
*Variant rs429358*								
Father	40–75	73,100	33,393	0.0752	1.0588	0.0212	7.03 × 10^−3^	−0.30
Father	75+	35,778	24,086	0.1147	1.1216	0.0252	5.34 × 10^−6^	−0.65
Mother	40–75	75,576	20,780	0.0858	1.0896	0.0268	0.0013	−0.19
Mother	75+	46,365	25,846	0.1847	1.2028	0.0241	1.72 × 10^−14^	−1.13
								
*Variant rs10519203*								
Father	40–75	111,025	51,984	0.0924	1.0968	0.0131	1.66 × 10^−12^	−0.47
Father	75+	53,244	36,139	0.0321	1.0327	0.0158	0.043	−0.16
Mother	40–75	115,003	32,758	0.0565	1.0581	0.0165	6.24 × 10^−4^	−0.26
Mother	75+	69,620	39,201	0.0292	1.0296	0.0152	0.055	−0.23

*P* values are given for the discovery analysis of parental allelic dose effect for the sentinel SNP at each locus, separately for maternal and paternal lifespans and younger (40–75) and older (75+) ages. Beta is the effect size from the full Cox model, HR is hazard ratio, years gives the reduction in lifespan in years for a (parental generation) individual carrying one copy of the variant.
