# Supplementary material for: Variants near CHRNA3/5 and APOE have age- and sex-related effects on human lifespan
Source: Nat Commun. 2016 Mar 31;7:11174. doi: 10.1038/ncomms11174 (PMC5438072; doi:10.1038/ncomms11174)
Supplement: Supplementary Information — Supplementary Figure 1, Supplementary Tables 1-3 [file ncomms11174-s1.pdf]

**Supplementary Figure 1. Quantile-quantile plots for genome-wide association**. Q-Q plots are presented from the discovery analysis for (a) paternal lifespan (b) maternal lifespan, (c) meta-analysis of mothers and fathers, and (d) meta-analysis of mothers and fathers conditioned on the most significant *APOE* and *CHRNA3/5* region SNPs.

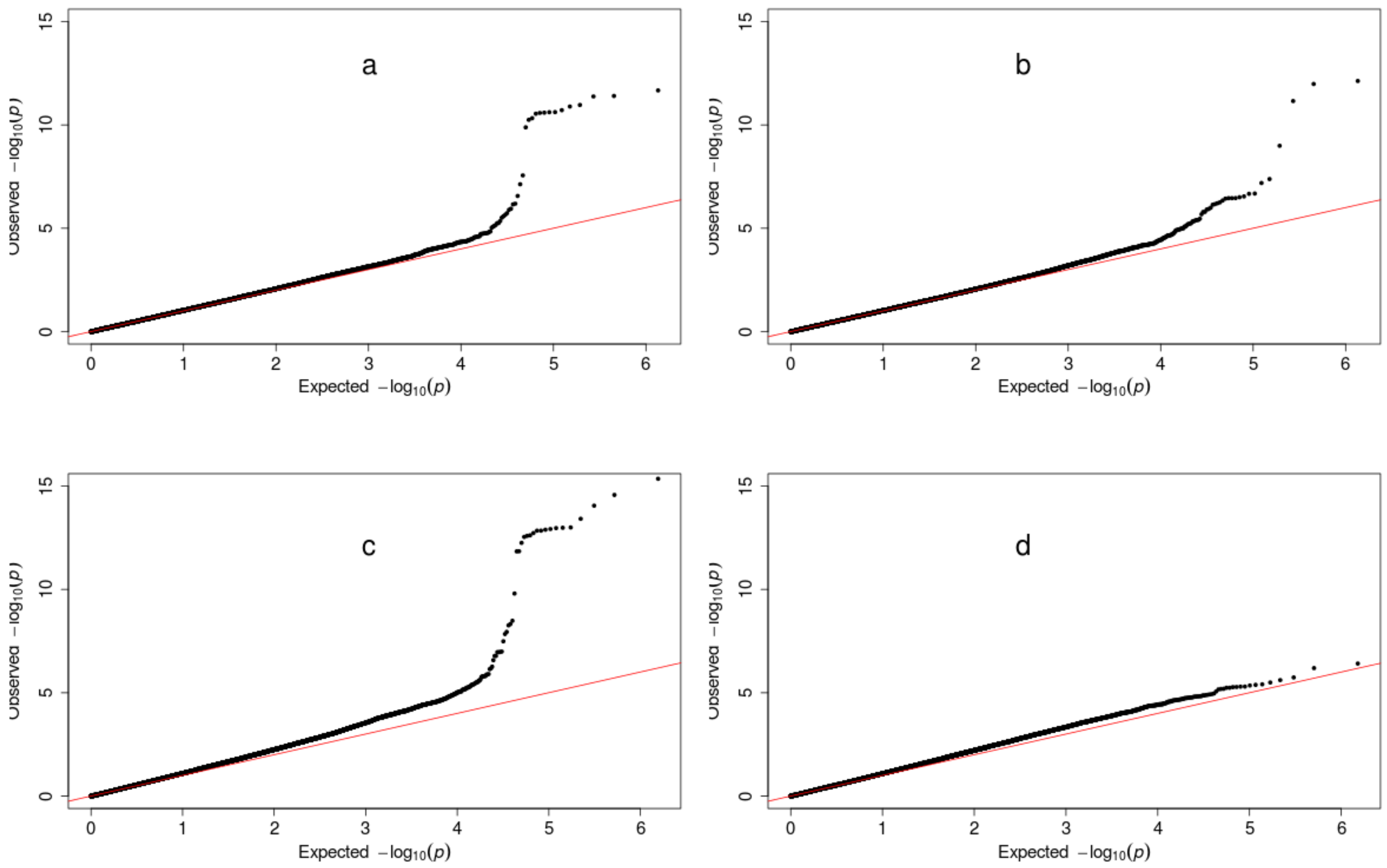

**Supplementary Table 1. Most significant SNPs in discovery genome-wide association.** All associations below P = 10-6 are shown. Positions use GRCh37, rsid are the dbSNP rs numbers, maf is minor allele frequency in UKB genetically British sample, beta is the estimated effect size on Martingale residuals of the Cox model, SE is the standard error of the effect sizes. The sentinel SNPs are italicised.

| Population | Chr | SNP | Position | Eff allele | Alt allele | N | Beta | SE | P | MAF |
|---|---|---|---|---|---|---|---|---|---|---|
| Fathers | 15 | rs11858836 | 78783277 | A | G | 110627 | 0.0485 | 0.00905 | 8.33E-08 | 0.317 |
| | 15 | rs9788721 | 78802869 | C | T | 111026 | 0.0573 | 0.00897 | 1.66E-10 | 0.343 |
| | 15 | rs8034191 | 78806023 | C | T | 111130 | 0.0609 | 0.00900 | 1.33E-11 | 0.333 |
| | *15* | *rs10519203* | *78814046* | *G* | *A* | *111025* | *0.0616* | *0.00899* | *7.50E-12* | *0.337* |
| | 15 | rs931794 | 78826180 | G | A | 111029 | 0.0606 | 0.00899 | 1.56E-11 | 0.337 |
| | 15 | rs55853698 | 78857939 | G | T | 111010 | 0.0593 | 0.00900 | 4.45E-11 | 0.331 |
| | 15 | rs55781567 | 78857986 | G | C | 110974 | 0.0596 | 0.00900 | 3.69E-11 | 0.335 |
| | 15 | rs17486278 | 78867482 | C | A | 111113 | 0.0588 | 0.00903 | 7.13E-11 | 0.332 |
| | 15 | rs72740964 | 78868636 | A | G | 110237 | 0.0569 | 0.00909 | 3.85E-10 | 0.326 |
| | 15 | rs7180002 | 78873993 | T | A | 110759 | 0.0586 | 0.00905 | 9.24E-11 | 0.328 |
| | 15 | rs951266 | 78878541 | A | G | 110991 | 0.0585 | 0.00903 | 9.44E-11 | 0.328 |
| | 15 | rs16969968 | 78882925 | A | G | 111072 | 0.0583 | 0.00903 | 1.03E-10 | 0.327 |
| | 15 | rs1051730 | 78894339 | A | G | 111004 | 0.0586 | 0.00903 | 8.76E-11 | 0.328 |
| | 15 | rs1317286 | 78896129 | G | A | 111039 | 0.0573 | 0.00902 | 2.06E-10 | 0.333 |
| | 15 | rs8040868 | 78911181 | C | T | 110975 | 0.0457 | 0.00870 | 1.47E-07 | 0.393 |
| | 15 | rs4243084 | 78911672 | C | G | 110950 | 0.0582 | 0.00900 | 9.82E-11 | 0.336 |
| Mothers | 19 | rs283815 | 45390333 | G | A | 75177 | 0.0622 | 0.01242 | 5.55E-07 | 0.222 |
| | 19 | rs6857 | 45392254 | T | C | 75624 | 0.0827 | 0.01354 | 1.01E-09 | 0.166 |
| | 19 | rs71352238 | 45394336 | C | T | 67708 | 0.0821 | 0.01518 | 6.42E-08 | 0.147 |
| | 19 | rs157581 | 45395714 | C | T | 115072 | 0.0557 | 0.01015 | 4.14E-08 | 0.218 |
| | 19 | rs34404554 | 45395909 | G | C | 75724 | 0.0717 | 0.01444 | 6.76E-07 | 0.143 |
| | 19 | rs157582 | 45396219 | T | C | 75656 | 0.0630 | 0.01239 | 3.68E-07 | 0.220 |
| | 19 | rs59007384 | 45396665 | T | G | 75667 | 0.0652 | 0.01256 | 2.15E-07 | 0.208 |
| | 19 | rs769449 | 45410002 | A | G | 114421 | 0.0866 | 0.01263 | 6.97E-12 | 0.121 |
| | *19* | *rs429358* | *45411941* | *C* | *T* | *75576* | *0.1008* | *0.01406* | *7.45E-13* | *0.153* |

| | | | | | | | | | | |
|---|---|---|---|---|---|---|---|---|---|---|
| | 19 | rs4420638 | 45422946 | G | A | 111666 | 0.0768 | 0.01078 | 1.04E-12 | 0.187 |
| Both | 4 | rs72631060 | 60247508 | T | C | | -0.0878 | | 3.33E-07 | |
| | 5 | rs17844309 | 140214995 | A | G | | -0.1886 | | 9.50E-07 | |
| | 6 | rs74617384 | 160997118 | T | A | | 0.0588 | | 9.83E-08 | |
| | 15 | rs11858836 | 78783277 | A | G | | 0.0350 | | 3.28E-08 | |
| | 15 | rs9788721 | 78802869 | C | T | | 0.0436 | | 3.91E-12 | |
| | 15 | rs8034191 | 78806023 | C | T | | 0.0460 | | 2.82E-13 | |
| | *15* | *rs10519203* | *78814046* | *G* | *A* | | *0.0468* | | *1.03E-13* | |
| | 15 | rs931794 | 78826180 | G | A | | 0.0460 | | 2.80E-13 | |
| | 15 | rs55853698 | 78857939 | G | T | | 0.0452 | | 7.62E-13 | |
| | 15 | rs55781567 | 78857986 | G | C | | 0.0454 | | 6.72E-13 | |
| | 15 | rs17486278 | 78867482 | C | A | | 0.0460 | | 3.69E-13 | |
| | 15 | rs72740964 | 78868636 | A | G | | 0.0464 | | 3.53E-13 | |
| | 15 | rs7180002 | 78873993 | T | A | | 0.0462 | | 3.39E-13 | |
| | 15 | rs951266 | 78878541 | A | G | | 0.0462 | | 2.94E-13 | |
| | 15 | rs16969968 | 78882925 | A | G | | 0.0456 | | 5.29E-13 | |
| | 15 | rs578776 | 78888400 | A | G | | -0.0348 | | 1.73E-07 | |
| | 15 | rs1051730 | 78894339 | A | G | | 0.0454 | | 6.73E-13 | |
| | 15 | rs1317286 | 78896129 | G | A | | 0.0446 | | 1.58E-12 | |
| | 15 | rs12914385 | 78898723 | T | C | | 0.0350 | | 9.94E-09 | |
| | 15 | rs8040868 | 78911181 | C | T | | 0.0384 | | 2.85E-10 | |
| | 15 | rs4243084 | 78911672 | C | G | | 0.0456 | | 4.48E-13 | |
| | 19 | rs12972156 | 45387459 | G | C | | 0.0606 | | 6.37E-09 | |
| | 19 | rs34342646 | 45388130 | A | G | | 0.0536 | | 1.25E-07 | |
| | 19 | rs283815 | 45390333 | G | A | | 0.0450 | | 4.03E-07 | |
| | 19 | rs6857 | 45392254 | T | C | | 0.0686 | | 1.26E-12 | |
| | 19 | rs71352238 | 45394336 | C | T | | 0.0572 | | 1.39E-07 | |
| | 19 | rs157581 | 45395714 | C | T | | 0.0398 | | 3.69E-08 | |
| | 19 | rs34095326 | 45395844 | A | G | | 0.0598 | | 1.71E-07 | |
| | 19 | rs34404554 | 45395909 | G | C | | 0.0584 | | 1.49E-08 | |

| | | | | | | |
|---|---|---|---|---|---|---|
| 19 | rs157582 | 45396219 | T | C | 0.0442 | 6.00E-07 |
| 19 | rs59007384 | 45396665 | T | G | 0.0470 | 1.65E-07 |
| 19 | rs769449 | 45410002 | A | G | 0.0706 | 4.87E-15 |
| *19* | *rs429358* | *45411941* | *C* | *T* | *0.0824* | *2.47E-16* |
| 19 | rs4420638 | 45422946 | G | A | 0.0594 | 1.12E-14 |
| 21 | rs17275490 | 17143899 | G | A | 0.0904 | 5.16E-07 |

**Supplementary Table 2. Mean and maximum parent ages by status for 116,425 subjects in the discovery phase.** Max is maximum.

| Parent | Status | Number | Mean age | Max age |
|---|---|---|---|---|
| Father | Dead | 85,419 | 71.3 | 105 |
| Father | Alive | 25,915 | 77.9 | 102 |
| Mother | Dead | 69,990 | 74.9 | 107 |
| Mother | Alive | 45,333 | 78.5 | 105 |

**Supplementary Table 3. Flow and counts of participants and parents through phenotypic and genotypic quality control in UK Biobank.** Table 1 Lives shows the total parental lives for UK Biobank, summing genetically British, declared British and other origins and matches the totals for these categories for each SNP.

| | |
|---|---|
| **UK Biobank Participants** | **502,664** |
| Not yet genotyped | *349,932* |
| Available for quality control | 152,732 |
| Adopted | *2296* |
| No parent age >40 | *3278* |
| Age at death >115 | *2* |
| Vague or small ethnicity | *7065* |
| Missing covariate | *181* |
| **Participants available for analysis** | **139,910** |

| | **Mother** | **Father** | **Total** |
|---|---|---|---|
| Participants available for analysis | 139,910 | 139,910 | 279,820 |
| Parental data missing | *1374* | *6365* | *7739* |
| **Parents available for analysis** | **138,536** | **133,545** | **272,081** |
| Of which genotyped at SNP | Mother | Father | Table 1 Lives |
| rs429358 | 92,245 | 89,076 | **181,321** |
| rs10519203 | 138,290 | 133,310 | **271,600** |
